# A system for automatically extracting clinical events with temporal information

**DOI:** 10.1186/s12911-020-01208-9

**Published:** 2020-08-20

**Authors:** Zhijing Li, Chen Li, Yu Long, Xuan Wang

**Affiliations:** 1 School of Computer Science and Technology, Xi’an Jiaotong University, Xi’an, 710049 Shaanxi China; 2 Shaanxi Province Key Laboratory of Satellite and Terrestrial Network Tech. R&D, Xi’an Jiaotong University, Xi’an, 710049 Shaanxi China

**Keywords:** Clinical text mining, Event extraction, Temporal extraction, Relation extraction, Piecewise representation, Attention mechanism

## Abstract

**Background:**

The popularization of health and medical informatics yields huge amounts of data. Extracting clinical events on a temporal course is the foundation of enabling advanced applications and research. It is a structure of presenting information in chronological order. Manual extraction would be extremely challenging due to the quantity and complexity of the records.

**Methods:**

We present an recurrent neural network- based architecture, which is able to automatically extract clinical event expressions along with each event’s temporal information. The system is built upon the attention-based and recursive neural networks and introduce a piecewise representation (we divide the input sentences into three pieces to better utilize the information in the sentences), incorporates semantic information by utilizing word representations obtained from BioASQ and Wikipedia.

**Results:**

The system is evaluated on the THYME corpus, a set of manually annotated clinical records from Mayo Clinic. In order to further verify the effectiveness of the system, the system is also evaluated on the TimeBank _Dense corpus. The experiments demonstrate that the system outperforms the current state-of-the-art models. The system also supports domain adaptation, i.e., the system may be used in brain cancer data while its model is trained in colon cancer data.

**Conclusion:**

Our system extracts temporal expressions, event expressions and link them according to actually occurring sequence, which may structure the key information from complicated unstructured clinical records. Furthermore, we demonstrate that combining the piecewise representation method with attention mechanism can capture more complete features. The system is flexible and can be extended to handle other document types.

## Background

Precision medicine is an emerging approach for disease treatment and prevention. It becomes the whole world biomedicine domain the research hot spot, which needs the support of biomedical methods, e.g. data mining. It associates with key information extracted from clinical records, e.g. symptoms over a disease course. The associations are often statistically concluded from the evidence collected from the clinical records [1]. The medical big data mostly exists in an unstructured form, e.g. text, which could store useful information very well. Aligning biomedical events in clinical data along the events’ actually occurring time is a meaningful and efficient way of structuring such complex data. The result may assist auxiliary diagnosis, treatment scheme determination, epidemic prediction and side effect discovery etc.

**Fig. 1:**
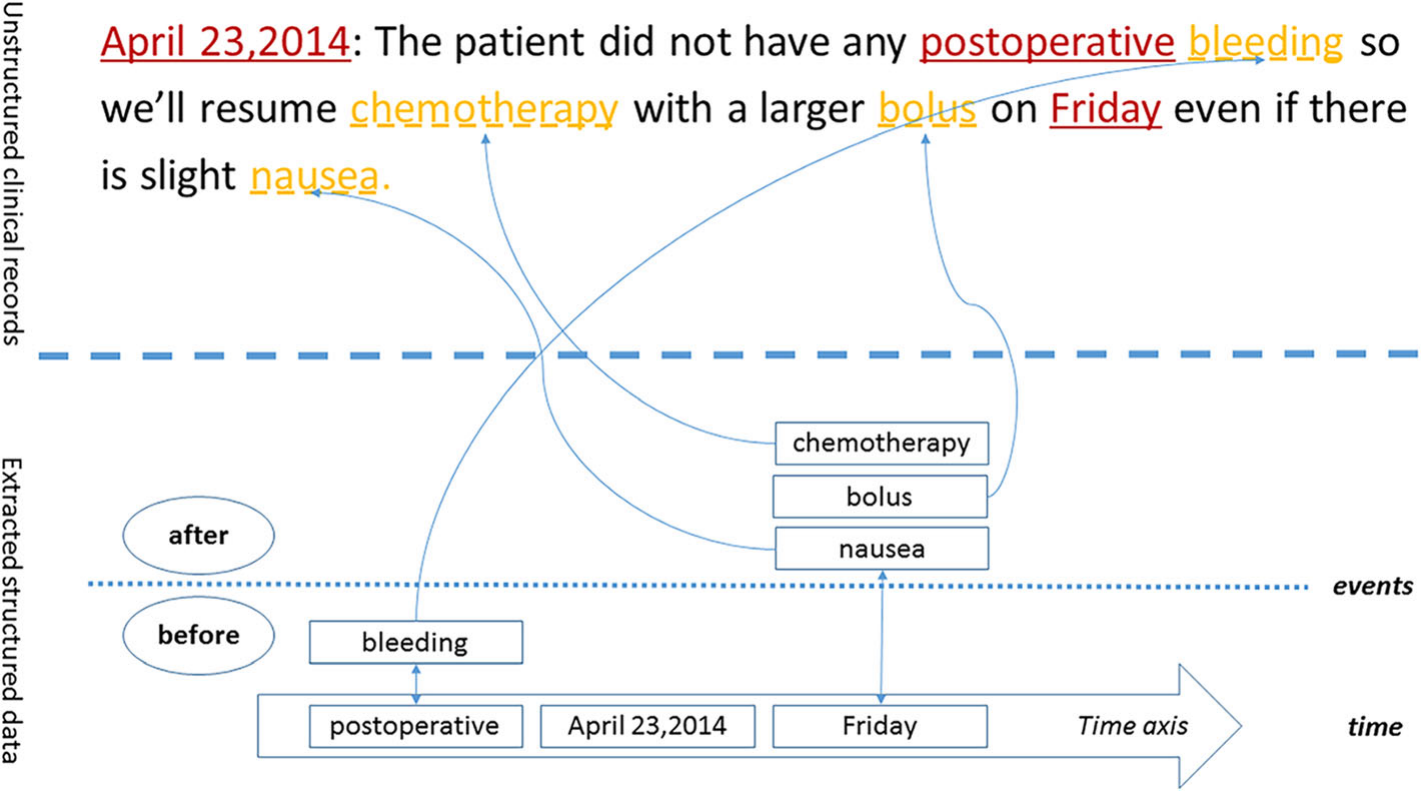
The example of the medical information extraction. The texts marked by the underscores, “___”, are the temporal expressions; the texts marked by the dash lines, “_ _ _”, are the event expressions

Many works have been devoted in the study of application in the medical era. However, large data analysis of medical treatment needs to map the corresponding medical events in the clinical records; the medical events with temporal information are very useful in medical era. These efforts will become the foundation of understanding disease, facilitating the analysis of large medical data as well. For example, the clinical record in Fig. 1 may be presented in a structured manner as the occurring events along with temporal information. It is easier for understanding the events and corresponding time point. For example, using the time point ‘April 23, 2014’ as a reference, the entity ‘bleeding’ is before the time point and the entity ‘bolus’, ‘chemotherapy’ and ‘nausea’ is after the time point. In such case, the actual events and their occurring consequence becomes clear at a glance.

In this paper, we present a novel system, which is built upon deep neural networks to automatically extract event expressions and their related temporal expressions from clinical records. The system has been evaluated on the Temporal Histories of Your Medical Event (THYME) corpus; a corpus developed by a number of professionals [2]. According to characteristics of the corpus, clinical data contains very long sentences that will undoubtedly increase the difficulty of processing. Therefore, we do not simply use neural networks, we want to make full use of the contextual information. Our proposed method organically combines piecewise representation and attention mechanism by a recurrent neural network (RNN), and achieves the state-of-the-art performance. The results show improvements in automatic extraction of clinical event expressions along with each event’s temporal information.

## Related work

Extracting clinical events along with temporal information is a complicated task and the existing systems often accommodate several independent components, each of which retrieves different parts, e.g. events and time, and assemble them together. Each component may use a set of hand crafted rules or be based on a pretrained ML model. Velupillai et al. develop the BluLab system include the ClearTK support vector machine and conditional random fields classification approach, and get the first place in SemEval-2015 Task 6: Clinical TempEval [3]. MacAvaney et al. present the system GUIR, include conditional random fields and decision tree ensembles, using lexical, syntactic, semantic, distributional, and rule based features [4]. GUIR receive the best score in SemEval-2017 Task 12: Clinical TempEval in the way of temporal expressions extraction. Tourille et al. use a neural network based approach and achieve good performance for both event and relation extraction in SemEval-2017 Task 12: Clinical TempEval [5]. Lin et al. propose a recurrent neural network with multiple semantically heterogeneous embeddings within a self-training framework for clinical temporal relation extraction task [6]. They achieve good results for both in- and cross-domain.

After event and temporal expression extraction, assigning each event with the right temporal expression involves more complicated process. Some systems match event with temporal expression by a set of syntactic rules crafted by experts. Wang et al. use syntactic rule-based method for automatic pathway relation information extraction from biomedical literature [7]. These methods are fast but not flexible enough. Some existing methods for medical relation information extraction are based on machine learning (ML) models [8–15].

Conditional random field (CRF) [16] and supper vector machine (SVM) [17] are often used in the task of relation extraction. Lu Liu et al. propose an SVM model to extract the relations between the potential named entity pairs [18]. Finkel et al. propose a CRF-based information extraction system to determine relationships [19]. Deep Learning revives the popularity of neural networks, which can learn effective relation features from the given sentences without complicated feature engineering. Socher et al. [8] is the first work that employs an RNN model to classify relation. One early work proposed by Luo [9] is based on a recurrent neural network and able to classify relations from clinical notes. Compared with the rule based methods, these methods are more flexible. Neural network based methods take less time by quickly screening out the most unlikely candidate entity-pairs. Therefore, some approaches attempt to combine both. Hence, we think RNN is a good choice and we adopt the RNN system.

In recent years, attention mechanism has been widely used in various tasks of NLP based on in-depth learning. Li et al. [20] propose a model that combines a bidirectional long short-term memory network with a multi-attention mechanism for relation extraction. Zhou et al. [21] propose the Attention-Based Bidirectional Long Short-Term Memory Networks (Att-BLSTM) for relation classification. And the model results outperforms most of the existing methods with only word vectors on the SemEval-2010 relation classification task. So in this paper we also introduce the attention mechanism.

## Methodology

The system consists of three components. The first component extracts temporal expressions. Temporal expressions: enable events to be chronologically annotated. The second component identifies the relevant medical events. Event expressions: any situation relevant to the patient’s clinical timeline. The third component detects the relations between the events and the temporal expressions.

Annotating clinical records are very expensive. Frequently, only a data of disease specific type is available. Besides the regular ML-based extraction, we introduce domain adaption to allow the system to be able to extract the information from one type of disease, e.g. brain cancer, while it is trained on another type, e.g. colon cancer.

**Fig. 2:**
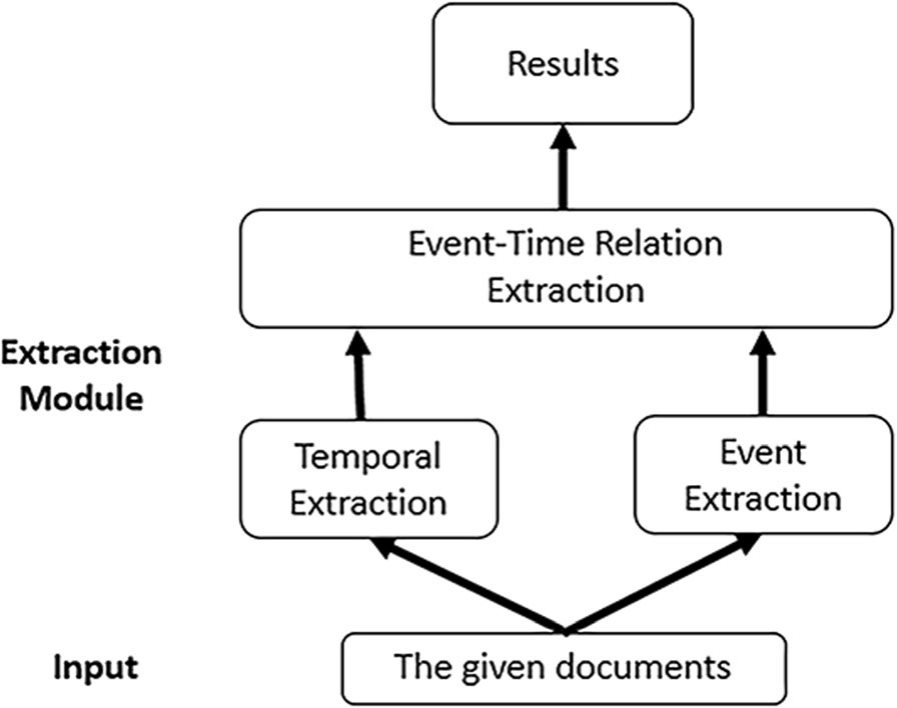
The data-processing pipeline of the system. We first extract temporal expressions and event expressions respectively from medical records, then extract the relations between them

We show the pipeline of our system in Fig. 2. The system is built upon an annotating pipeline adopting the Unstructured Information Management Architecture (UIMA) framework. The preprocessing includes tokenization, part-of-speech tagging and lemmatization, which used the Stanford coreNLP toolkit [22]. In both time and event extractions, spans of time and event expressions are represented by the offsets in texts. The automatic annotations of event expressions, temporal expressions and their relations are based on three RNN models utilizing lexical, syntactic and semantic features [23–30]. At the core of deep learning techniques for NLP lies the vector based word representation, which maps words to an n-dimensional space. For the choice of the word embeddings corpus, we do some research work. Only 70% entities (time and event expressions) in the clinical records can be found in the Wikipedia. In comparison, the BioASQ corpus which is full of biomedical information contains more than 90% of the entities. We use word embeddings from the European project BioASQ obtained by using word2vec [31] on 10,876,004 PubMed abstracts [32, 33] and include the vectors of 1,701,632 distinct words. Each word is represented as a 200-dimensional vector. If a word could not be found in the BioASQ corpus, the embedding is generated from Wikipedia by word2vec (Mikolov et al., 2013) as a complement [34, 35].

The internal state of RNN can demonstrate dynamic timing behavior [36]. The hidden state vector can be computed by the following formula.

$$ {h}_t=\mathrm{F}\left(\mathrm{W}{h}_{t-1}+\mathrm{U}{x}_t\right) $$

In this formula, *x*_*t*_ is the input, *h*_*t*_ is the hidden state, U and W are the weight coefficients, F is the nonlinear function such as tanh or ReLu.

### Extraction of temporal expressions and event expressions

Independent models are trained for the extractions of temporal expressions and event expressions. Figure 1 shows the infrastructure of the system. There are two forms of temporal expressions. One is numeric temporal expressions (e.g. 12:30, 8:40 etc.), and the other is casual temporal expressions (e.g. 1 day, 2 weeks, during a period etc.). Firstly, we generalize all the numeric temporal expressions into 00:00. For example, both 12:30 and 8:40 become 00:00, which can be easily recognized by the regular expression. The regular expression is used due to the characteristics of the data. The numeric temporal expressions are not well recognized by RNN if we do not use it. Secondly, the casual temporal expressions are recognized by a RNN model. The casual temporal expressions in the training set are tagged to represent the token’s position in a particular expression [37]. There are four tags in our proposed method including “B”, “I”, “O”, and “E” which state that the token is at the beginning, on the inside, on the outside, or at the end of the entity respectively.

**Fig. 3:**
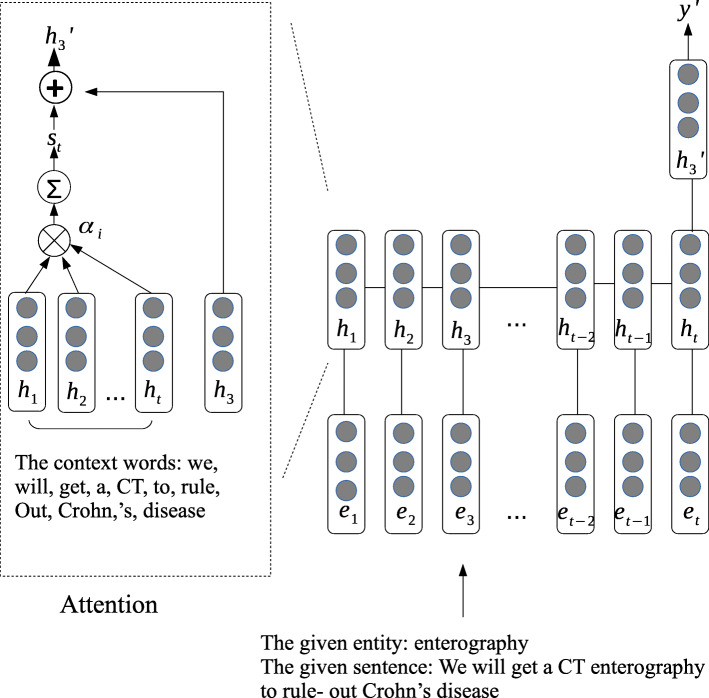
Architecture of the RNN model for the extraction of temporal expressions and event expressions. We propose the attention-based RNN model to do the entity extraction. On the left side of the figure is the details of the attention mechanism, the right part of the figure is the RNN model

In this section, we propose the system (ARNN), which is based on a recurrent neural network combining the attention mechanism. We need to predict the token’s position tag of each word before we can train the ARNN model to predict the type of each temporal expression. We treat each temporal expression as an entity and each entity is treated as a unit input, we use the average value of all the word embeddings of an entity in the next process. The network in the Fig. 3 shows the flow chart of ARNN network to predict the type of the entity. There is an example sentence from the corpus “We will get a CT enterography to rule- out Crohn’s disease”. In this case, the given entity is “enterography”, and the context words are “we, will, get, a, CT, to, rule, out, Crohn, disease”. In order to better apply the context information, we employ the attention mechanism to learn the weighted score of each context word related to the given entity.

The higher weight, the higher semantic is bound up with the given entity.

$$ {\alpha}_i\propto \exp \left({v}_i^T{U}_v{h}_t\right) $$

*Uv* is the parameter that has to be learned. From Fig. 3 we can see that *s*_*t*_ is the state vector that integrates the other context words information with the given entity at time t. *s*_*t*_ can be computed as:

$$ {s}_t=\sum \limits_{i\in h}{\alpha}_i{h}_i $$

We combine *s*_*t*_ and *h*_3_ to obtain ${h}_3^{\prime }$, which can represent the given entity.

$$ {h}_3^{\prime }={h}_3+{s}_t $$

In this process, the prediction of entity’s type will be predicted from the given input entity vector. The method of using regular expression are also used to match the missing temporal expressions (e.g. 2010-12-23).

Similar to temporal expressions extraction, the event expressions extraction is built on another RNN. Unlike time expressions, event expressions are all single words, there is no need to sign each token’s position. We use the softmax classifier to predict the label y^′^ of the temporal and event expressions. The state vector ${h}_3^{\prime }$ is used as input, and y^′^ could be computed by:

$$ {\displaystyle \begin{array}{c}{p}_y= softmax\left(W{h}_3^{\prime}\right)\\ {}{\mathrm{y}}^{\prime }=\arg \underset{y}{\max }{p}_y\end{array}} $$

### Event-time relation (ER)

ER extraction is the most important task in this paper. In this section, we propose the novel system (APRNN), which is based on a recurrent neural network combining the attention mechanism and the piecewise representation. The event-time relation are regarded as a classification problem, it is divided into four categories based on some well-known communities such as SemEval or BioNLP. The event time relation associates the identified event expressions and temporal expressions, and indeed indicates the WHAT and WHEN of a medical event in clinical records. The four types are before, after, before/overlap and overlap.

#### Piecewise representation

**Fig. 4:**
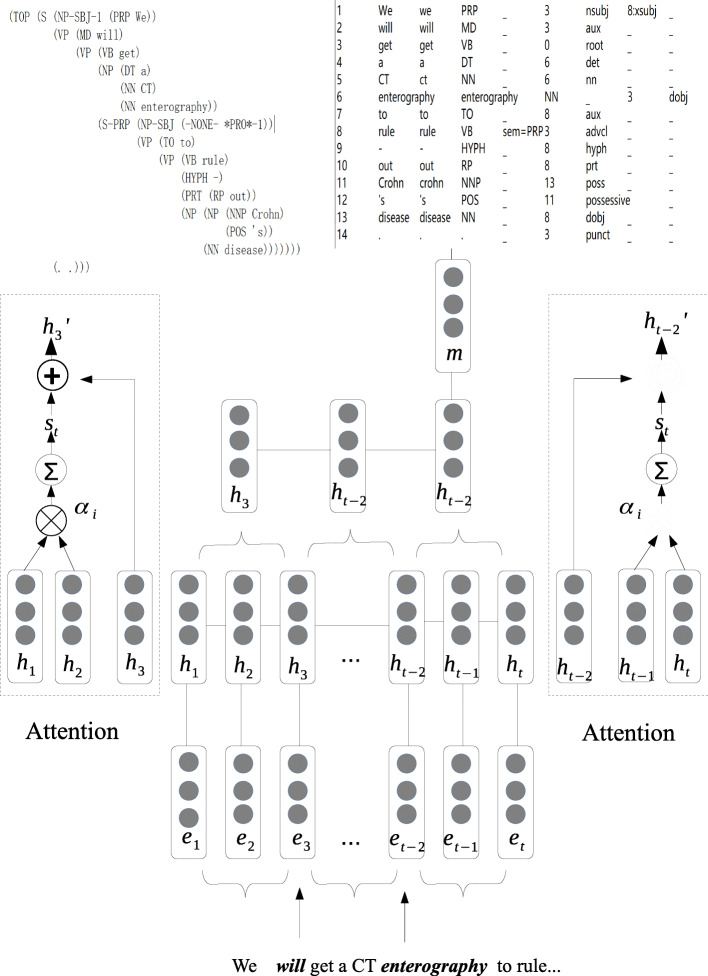
The flow of our proposed model (APRNN). On the left side of the figure is the details of the attention mechanism, the right part of the figure is the RNN model, which is divided into three parts

The dependency parsing of each sentence has been obtained by utilizing Stanford coreNLP toolkit. The previously trained word embeddings, which represent each word by a 200-dimension word vector, and the shortest syntactic paths are fed into the RNN model of ER extraction. The word embeddings and shortest syntactic path are used as features. The information of the shortest syntactic path used includes the words, the POSs and the length. We add all these vectors as the entity feature. After adding up, we still get a 200-dimensional vector for each entity. If the entity include several tokens (e.g. temporal expressions), we add the vectors of each token and get the average vector as the entity vector. The whole article is divided into sentences as input units. We extract all entity pairs based on the annotations. Given a sentence *X* = (*x*_*1*_, *x*_*2*_, …, *x*_*T*_), the words are projected into a sequence of word vectors, denoted by (*e*_*1*_, *e*_*2*_, ..., *e*_*T*_) where T is the number of words. In this part, we would like to introduce the piecewise representation. In other words, the input sentence is divided into three parts according to the entity pair, we call this process piecewise representation. The purpose of piecewise representation is to better use of the context information. As shown in Fig. 4, the example sentence is divided into three parts according to the entity pair (will, enterography). The whole sentence is “We will get a CT enterography to rule-out Crohn’s, disease.” The first part is the sequence before the entity pair (we), the second part is the sequence between the entity pair (get, a, CT), and the third part is the sequence after the entity pair (to, rule…). There are reasons for segmenting the sentence. The first one is that in many cases, some studies may choose the sentence between the entity pair as input instead of using the whole sentence [38]. Nevertheless, this can miss some information, and some of them may be useful. Only several words cannot supply enough context information for extracting features. Segmented sentences can be used to extract the effective information to the greatest extent of each sequence and avoid the absence of contextual information. Another reason comes from the network and our corpus. In the corpus, the longest sentence contains 235 words. However, the average length of all sentences has 18 words. With the RNN structure, since the information of a sentence is learned word by word, the feature vector produced at the end of the sentence actually represents the entire sentence. Although RNN has the memory in learning process, but the memory time is not long. Accumulation by recurrent connections tends to forget long-term information quickly, and the feature vector at the end of the sentence is hard to carry the information of early steps in model training. There are many long-distance sentences (more than 100 words) in the training data, so the piecewise representation can help the system better use the information of the sentence. The syntactic analysis and POS information of the example sentence are also shown in the Fig. 4.

#### Model

The ER model contained three parts. The first component, the feature layer; The second component, the hidden layer, catch the information of word sequence and produces word-level features’ representations and then merges word-level features into a sentence-level feature vector, by selecting the most valuable feature information among all the word-level features. We show the whole process in the Fig. 4. In this part, we propose the attention mechanism to obtain the representation of the sentence. Not all the words in the context describe the ER relation, each word in the context has different effects on the given entity pair. Therefore, we introduce the attention mechanism to learn the weighted score of each context word related to the entity pair. For the first part, the attention mechanism is used to screen the most useful information. We use the bilinear operator to compute the attention weight *α*_*i*_ for each vector (*h*_1_ *and h*_2_), to reflect how the information relevant to the first entity in the entity pair (the current state *h*_3_). The calculation method of *α*_*i*_ and *s*_*t*_ is the same as the description in the “Extraction of temporal expressions and event expressions” section.

We combine *s*_*t*_ and *h*_3_ to obtain ${h}_3^{\prime }$, which can represent the sequence before the entity pair. For the second part, we choose the state of the last entity in the sequence (*h*_*t* − 2_) to represent the whole sequence. For the third part, the same method is used as the first part. We also use the same attention mechanism to obtain the representation of the sequence (${h}_{t-2}^{\prime }$). The single sentence-level feature vector need to be obtained to represent the entire sentence for the relation classification. We introduce the max-pooling approach as in CNN models to obtain the single sentence-level feature vector [25]. The max-pooling is formulated as follows:

$$ \mathrm{m}=\underset{t}{\max}\left\{{h}_t\right\} $$

Next, the sentence-level feature vector m is passed to the output layer. Furthermore, the output layer has 4 classes. We use the softmax classifier to predict the label y^′^ from a set of labels Y from the sentence. The state vector m is used as input, therefore y^′^ could be computed by:

$$ {\displaystyle \begin{array}{c}{p}_y= softmax(Wm)\\ {}{\mathrm{y}}^{\prime }=\arg \underset{y}{\max }{p}_y\end{array}} $$

**Fig. 5:**
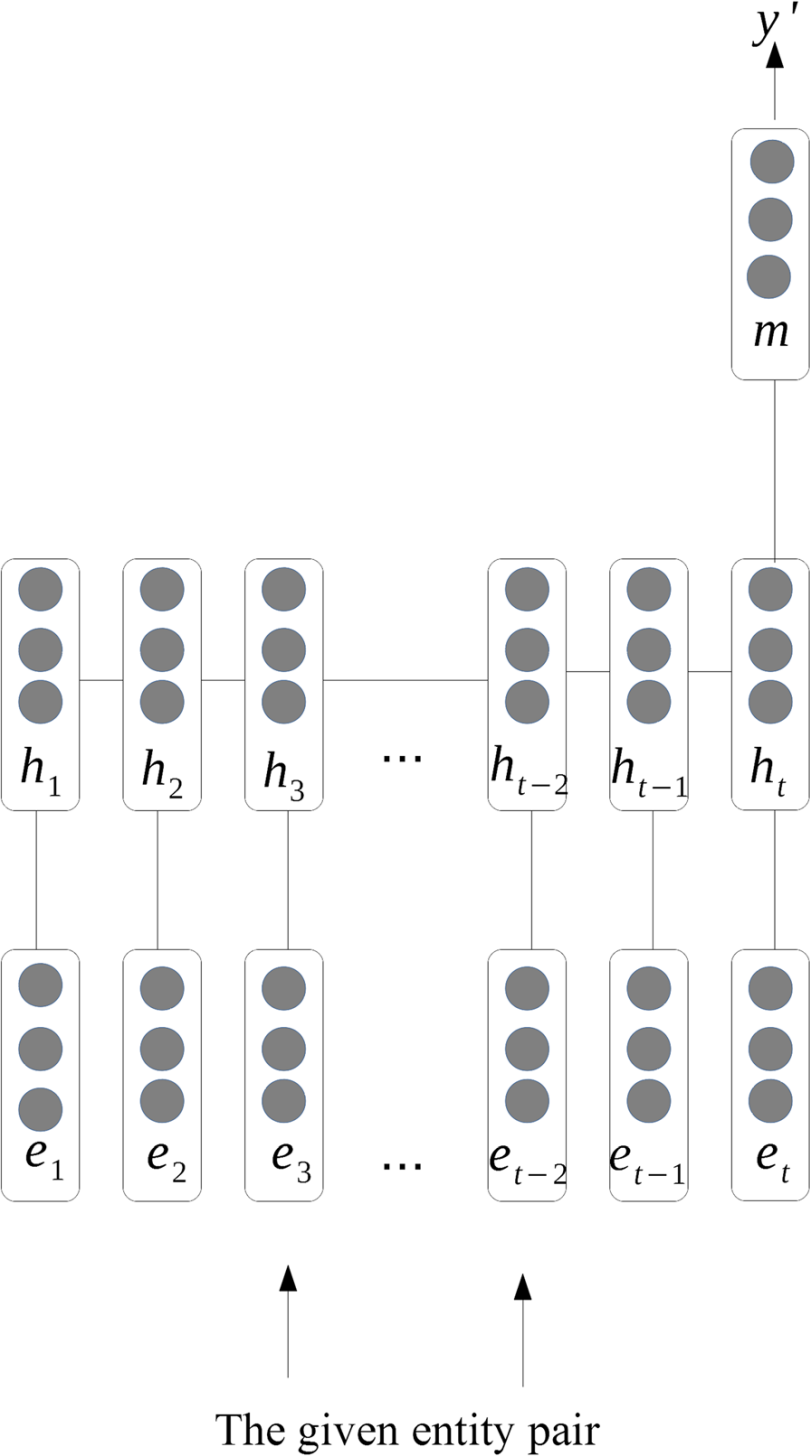
The flow of the Comparison system. This is a common RNN model

In addition, there are two settings in ER extraction in order to better compare our approach. The first is based on our proposed method, the second is only utilize the RNN network without any Piecewise representation or attention mechanism as shows in Fig. 5.

## Experiment and results

### Data

The major medical data are the data of medical institutions’ diagnosis and treatment, collection of massive clinical data and laboratory data produced every day at all levels of hospital.

A golden annotated corpus, marked up with temporal expressions, events and relationship between them is needed to allow us to evaluate by our methods. The THYME corpus, which has been used since 2011 [39], is one of the suitable corpora, consisting of clinical and pathological notes of patients with colon cancer and brain cancer from Mayo Clinic. Unlike other datasets, the events in this dataset are all single words, which are very suitable for our system. The notes are manually annotated by the THYME project (thyme.healthnlp.org) using an extension of ISOTimeML for the annotation of temporal expressions, events and temporal relations [40]. 50% of the corpus is used for training, 25% is for development and 25% is for testing. The development set is used for optimizing learning parameters, then combine it with the training set to build the system used for reporting results. Table 1 shows the distribution of the THYME corpus. The colon cancer data are used as training data and are tested on both colon cancer and brain cancer data, to demonstrate its effectiveness with or without domain adaptation, and this can reflect that our approach is not limited to a particular field. In evaluation, all methods have access to the same set of training and testing data.

**Table 1:** The distribution of the THYME corpus. In this table, we show the different types of data in the corpus

Data	Colon cancer Train Dev Test	Brain cancer Train Test
**Document**	293,143,141	30,148
**Temporal expressions**	3833 2078 1952	3,501,552
**Event expressions**	38,890 20,974 18,990	2557 11,510
**ER**	11,150 6163 5894	6,241,759

### Results

The method has been evaluated on both colon cancer data and brain cancer data to demonstrate the effectiveness with or without domain adaptation. In order to do better research, several methods are used to de the entity extraction.

Six methods of temporal expression extraction. 1) a rule-based method; 2) a system based on CRF; 3) a system based on general RNN without any attention mechanism or context (RNN); 4) a system based on RNN with easy attention mechanism but without any context words (RNN-att); 5) our proposed method, a RNN system with attention mechanism and context words; 6) a system combines the CRF and RNN network. All the results are compared (part 1 in Tables 2 and 3). For the rule-based methods, firstly, we find all the prepositions, according to our experience and experimental statistics; we extract five tokens behind their own prepositions. Through careful observation of data, we found that many time expressions always show up behind a preposition, we then judge whether those five words are related to time expressions. We define a time dictionary to list the words which we think can be a part of the time expressions, like “month”, “week”, “day”, “hour”, “May”, “Monday”, “morning”, “once” and so on. Next, we contrast the five tokens with time dictionary, and find whether it can represent a date or a precise time. Finally, we extract all the continuous tokens that we thought may relate to the time expressions (if there is a definite article before those tokens, extract it as well). There exist some expressions do not after a preposition and only contain one word and most of them have the same prefix like “pre”, “post”, “peri”. So we use this prefix rule to find the remain expressions. The major feature we used for training the CRF and SVM classifier is simple lexical features (word embeddings, part-of-speech tag, numeric type, capital type, lower case). BluLab: run 1–3 and GUIR system are the previously best system mentioned in the section two. These results are shown in Tables 2 and 3 (part 2).

**Table 2:** The temporal expressions extraction results on colon cancer. The Part 1 shows the results of six different methods that we used to do the temporal expressions extraction; the Part 2 shows the result of the previously best system

	Method	P	R	F1
Part 1	Rule-based	0.412	0.594	0.486
	CRF	0.813	0.592	0.685
	RNN	0.662	0.629	0.645
	RNN-att	0.677	0.669	0.663
	ARNN	0.691	0.675	0.683
	CRF-ARNN	0.754	0.725	0.739
Part 2	BluLab: run 1–3	0.797	0.664	0.725

**Table 3:** The temporal expressions extraction results on brain cancer. We utilize 6 different methods to do the task, the results are shown in Part 1; the result of previously best system is shown in Part 2

	Method	P	R	F1
Part 1	Rule-based	0.33	0.52	0.41
	CRF	0.72	0.55	0.62
	RNN	0.63	0.57	0.60
	RNN-att	0.65	0.57	0.61
	ARNN	0.66	0.60	0.63
	CRF-ARNN	0.69	0.65	0.67
Part 2	GUIR	0.51	0.67	0.58

In both Tables 2 and 3, rule based methods achieve the lowest result. The recalls are relatively better than the precisions due to the well-defined dictionary. The error analysis shows that some “pre”, “post” and “peri”, are considered as time expressions while they should not be. Meanwhile, the rule-based method often mistakes two independent expressions as one if they are adjacent. In Table 2, the RNN system’s performances are lower than BluLab: run 1–3(a ClearTK SVM pipeline using mainly simple lexical features along with information from rule-based systems). The rule-based information is effective, but it has limitations; it can extract rules according to the characteristics of data. We do not add any rules to the RNN system. The observation on the error analysis shows that without any attention mechanism and context words, RNN is not very effective for similar combinations of numbers and letters (e.g. 20 h, 3 days etc.). Because the form of the corresponding word vectors are generated randomly, and the time series contains a large number of the above type, so the model cannot learn characteristics of time series, so it cannot be correctly extracted. After adding the attention mechanism and context words, the ARNN system achieve the relatively good results. Because of the good results of CRF, we combine the CRF with the ARNN and achieve the best result. From Table 3, we can see that the RNN outperforms the GUIR system, which is the current best system. (It is an ensemble of CRF and decision tree with lexical, syntactic, semantic, distributional, and rule-based features). The GUIR system can not extract the previously unseen or atypical date formats very well, it is obvious that their rules are not comprehensive enough. This problem also exists in RNN system, however, when adding the attention mechanism, it can extract more new and otherwise unknown formats. The ARNN and CRF-ARNN system achieve the best results. In this part, we have two test data sets, one is colon cancer, another one is brain caner. We trained all the models on the same training data and test them on two different test data sets. Except for the different test data, the parameters are exactly the same. The experimental results prove that our model is effective on other test data sets.

Meanwhile, five methods of event extraction. 1) a method based on SVM; 2) a system based on CRF; 3) a system based on general RNN without any attention mechanism or context (RNN); 4) a system based on RNN with easy attention mechanism but without any context words (RNN-att); 5) our proposed method, a RNN system with attention mechanism and context words. All the results are evaluated (part 1 in Tables 4 and 5). For event extraction, the SVM and CRF model obtain the relatively good results in colon data and perform poorly in brain colon data compared to the best system (LIMSI). However, RNN achieves preferably results in the two sets of test data, even higher than the best system (LIMSI). As shown in both Tables 4 and 5, when adding the attention mechanism and context words, the results are improved.

**Table 4:** The event extraction results on colon cancer. 5 different methods are utilized by us, all the results are shown in Par1; the Part 2 shows the result of the previously best system

	Method	P	R	F1
Part 1	SVM	0.860	0.843	0.851
	CRF	0.896	0.874	0.885
	RNN	0.893	0.897	0.893
	RNN-att	0.903	0.899	0.901
	ARNN	0.922	0.908	0.915
Part 2	BluLab: run 1–3	0.887	0.864	0.875

**Table 5:** The event extraction results on brain cancer. We adopt 5 methods to do the task, the results can be compared in Part 1; the result of the best system is shown in Part 2

	Method	P	R	F1
Part 1	SVM	0.55	0.69	0.61
	CRF	0.68	0.80	0.73
	RNN	0.75	0.83	0.77
	RNN-att	0.77	0.79	0.78
	ARNN	0.82	0.78	0.80
Part 2	LIMSI	0.69	0.85	0.76

As for the ER extraction, which is the key point of the paper. First, we compare our proposed model with the following methods: 1) a general RNN system without any attention mechanism or piecewise representation. We use the sentence between the entity pair as the input (RNN); 2) a general RNN system without any attention mechanism or piecewise representation. We use the whole sentence as the input (RNN-whole). We can see the results of RNN-whole is better than the results of RNN. It means that the sentence length can affect the performance of the system. Therefore, we use the sentence between the entity pair as the input for other system. 3) a general RNN system with attention mechanism but without piecewise representation (RNN-att). 4) a general RNN system without attention mechanism but with piecewise representation (RNN-pie). 5) our proposed system, APRNN, but only use the word embeddings trained from Wikipedia (APRNN-wiki). 6) our proposed system, APRNN, but only use the word embeddings trained from BioASQ (APRNN-BioASQ). 7) our proposed system, which is based on a recurrent neural network combining the attention mechanism and the piecewise representation. All these results are evaluated (part 1 in Tables 6 and 7). Except for model 5) and 6), the word embeddings for other models are from both Wikipedia and BioASQ. From the results, we can see that both attention mechanism and piecewise representation are useful. They can improve the results to some extent. We can directly compare the value of attention in two groups of experiments (result 1) and result 3); result 4) and result 7)). The result 3) and result 7), result 1) and result 4) can directly demonstrate the performance with and without segmentation. The difference between model 3) and 7) is that model 3) is missing the piecewise representation, and the difference between model 4) and 7) is without or with the attention mechanism. The result has been improved with the piecewise representation. The experiment 5) and 6) are about looking at the impact of word embeddings. The result 5) and result 6) show that different word embeddings can lead to different results. After combining the two corpus (Wikipedia and BioASQ), the results increase slightly (APRNN). Different factors that may affect the results are verified from experimental results, e.g. piecewise representation, attention mechanism, word embeddings. All these factors are utilized to make better use of contextual information.

**Table 6:** The ER classification results on colon cancer. Part 1 shows the results of the relevant methods we used; the other related works, which achieved the very good results are shown in Part 2

	Method	P	R	F1
Part 1	RNN	0.697	0.721	0.709
	RNN-whole	0.668	0.680	0.674
	RNN-att	0.719	0.715	0.717
	RNN-pie	0.717	0.709	0.713
	APRNN-wiki	0.727	0.717	0.722
	APRNN-BioASQ	0.731	0.723	0.727
	APRNN	0.733	0.711	0.729
Part 2	BluLab: run 1–3	0.712	0.693	0.702
	SVM	0.678	0.658	0.668
	Att-BLSTM	0.721	0.715	0.718

**Table 7:** The ER classification results on brain cancer. The results of our proposed methods are shown in Part 1; Part 2 shows the results of other related

	Method	P	R	F1
Part 1	RNN	0.61	0.59	0.60
	RNN (whole)	0.59	0.55	0.57
	RNN-att	0.61	0.61	0.61
	RNN-pie	0.62	0.60	0.61
	APRNN-wiki	0.63	0.61	0.62
	APRNN-BioASQ	0.64	0.62	0.63
	APRNN	0.65	0.59	0.63
Part 2	LIMSI	0.53	0.66	0.59
	SVM	0.57	0.53	0.55
	Att-BLSTM	0.63	0.61	0.62

We compare our work with other related work. The LIMSI system, which achieves the best score on the ER task in SemEval-2017 Task 12; Li, Rao and Zhang (2016) proposed the Litway, which is a system that has adopted a hybrid approach that uses the LibSVM classifier with a rule-based method for relation extraction [41]. They achieve the best score in the SeeDev task of BioNLP-ST 2016. Thus, we use their approach as a benchmark for our system. The BiLSTM-attention networks proposed by Zhou et al. [21] were chosen as another benchmarking model (Att-BLSTM), which outperforms most of the existing methods. They designed a bidirectional attention mechanism to extract word-level features from the sentence. The features for the attention-based model include word vectors and position indicators. For the sake of fairness, we also use the sentence between the entity pair as the input, but without the piecewise representation. The results are shown in the part 2 in Tables 6 and 7. The reported results involve reimplementation of all of the approaches. The SVM system can not get the whole information about the input sentence. The Att-BLSTM achieve the good results. However, we use the sentence between the entity pair as the input, the Att-BLSTM can not get the context information. The ER results show that APRNN has a higher performance in comparison with other systems. Both Tables 6 and 7 can show that our proposed model (APRNN) can effectively extract the biomedical entity relations. The APRNN model can better utilize the information in the context, which is extremely important for extracting biomedical entity relations.

To further verify our approach, we also validate our system on the data of TimeBank_Dense which is provided by The TempEval3 (TE3) workshop [42]. The TimeBank-Dense corpus contains 12,715 temporal relations over 36 documents taken from TimeBank 1.2. (22 documents training set, 5 documents development set and 9 documents test set). It is created to address the sparsity issue in the existing TimeML corpora. All pairs of events and time expressions are labeled. Some entity pairs may not in the same sentence. We still choose the sequence between the entity pair as the input sentence, the sequence before or after the two entities are used as the context words. We select several systems to do comparative experiments. Bethard propose the ClearTK system [43], which is the winner of TempEval-3. TempEval-3 use TimeBank documents, but remove a small portion of its events. Chambers et al. [44] propose the CAEVO system (a sieve-based architecture) on the TimeBank_Dense corpus; they achieve the state-of-art result and exceed other systems by a large margin. All the results are shown in Table 8. They make specific settings (e.g. rule-based classifiers) for the data. The Table 8 demonstrates that our proposed model (APRNN) has a better performance than the comparative models on the TimeBank_Dense corpus.

**Table 8:** The temporal relation classification results on TimeBank_Dense corpus

Method	P	R	F1
ClearTK	0.397	0.091	0.147
CAEVO	0.508	0.506	0.507
APRNN	0.511	0.507	0.509

Experimental results show that RNN model achieves good results in information extraction. However, the results based on APRNN obtain the highest value with or without domain adaptation. Experiments show that our system has a certain degree of universality; it is not limited to a specific data, but also suitable for other data.

## Discussion

The proposed model is capable of extracting structured information of clinical event expressions along with the corresponding temporal information. Our used corpus has its unique characteristics. The length of sentences in the corpus varies, and some sentences are too long. In order to make better use of the information in the sentences, we combine the RNN architecture with piecewise representation and attention mechanism. APRNN demonstrate very good performance on the test sets of colon cancer data and brain cancer data. The final results (in particular in Tables 6 and 7) show that the APRNN system can have a better performance than the system without the piecewise representation or attention mechanism. The elimination experiments can prove that these two factors (piecewise representation and attention mechanism) can influence the final results. However, only limited progress is made (The F1value is increased by 2% on colon cancer data and 3% on brain cancer data). Although the results of brain cancer data extraction are not bad, our system does not specifically incorporate domain adaptation methods. We think more domain adaptation methods can be used to further improve the results. In addition, our model does not add any position information or direction information, so if we need to handle the directionally relations, we need to extend the model further. Although our model is not perfect, it is based on the features of experimental data. The processes of different information extraction in current system is a pipeline. We will attempt to extract all information and structure clinical records in a joint-learning model to avoid erroneous propagation and possible neglect of contextual information.

We also utilize the attention-based RNN model to do the temporal and event expressions extraction. For event expressions, the THYME corpus is different from the general datasets, the events in this dataset are all single words. There is no need to sign each token’s position before the extraction. If we use other datasets, we need to label the data first. For temporal expressions extraction, although RNN has achieved better results than the best system on brain cancer data, the CRF model is even better. CRF has a good effect in detecting the mention tokens, RNN is better at classification. Our experimental results prove that combining CRF and RNN is a good idea.

In this paper, we mainly utilize the THYME corpus. However, the length of sentences in corpus is uneven; the longest sentence contains 235 words. For the ER extraction, the results of RNN-whole is better than the results of RNN. To a certain degree, it can prove that the sentence length can affect the performance of the system. For better verification, we have done further research. An investigation confirms that the performance declines when the sentences get longer. As the average length of all sentences has 18 words, the corpus is separated into three subsets for the analysis. The first subset contains those sentences with 18 or less words. The second subset contains the sentences with more than 18 words. The exceptionally long sentences, such as those with more than 100 words are considered separately. We use general RNN system to do the experiments about the influence of the sentence length on ER task. In Table 9, the performance is calculated towards the three subsets. It shows that better performances are achieved on the shorter sentences. This might be due to the long sentences often contain more than one event and the distances between events and times are relatively far. For this reason, we choose the sentence between the entity pair to do the experiments. Pointing at the THYME corpus, we propose the piecewise representation, which can improve the results to some extent. There are signs that our proposed method (combine the RNN architecture with piecewise representation and attention mechanism) can improve the processing effect of long sentences, capture features that are more complete.

**Table 9:** The ER classification results of different length of sentences

length of sentences	0–18	19–100	> 100
number of sentences	5311	2320	96
P	0.723	0.714	0.612
R	0.743	0.744	0.669
F	0.733	0.729	0.639

## Conclusion

In this paper, we present a novel system, which is able to automatically extract clinical event expressions along with each event’s temporal information. The system adopts RNN, and introduce the piecewise representation and attention mechanism meanwhile. Our system extracts temporal expressions, event expressions and link them according to actually occurring sequence, which may structure the key information from complicated unstructured clinical records. Both attention mechanism and piecewise representation can improve the results to some extent. The experimental results show that our whole system (RNN+ attention mechanism+ piecewise representation) achieves the state-of-the-art performances in the event and the general relation extraction with general applicability. The system is flexible and may be extended to handle other document types. According to the characteristics of different data sets, different designs should be made for the models. Full use of the information in the data can better get the desired results.

## Data Availability

The datasets obtained from the Mayo Clinic are identified. The datasets generated and analyzed during the current study are not publicly available due to the reason that we have signed a confidentiality agreement with Mayo hospital but are available from Semeval-2017 or Mayo hospital on reasonable request.

## Abbreviations

THYME: Temporal Histories of Your Medical Event
ML: Machine learning
CRF: Conditional random field
SVM: Supper vector machine
AttBLSTM: Attention-Based Bidirectional Long Short-Term Memory Networks
ER: Event-time relation
